# Duration of Neurocognitive Impairment With Medical Cannabis Use: A Scoping Review

**DOI:** 10.3389/fpsyt.2021.638962

**Published:** 2021-03-12

**Authors:** Lauren Eadie, Lindsay A. Lo, April Christiansen, Jeffrey R. Brubacher, Alasdair M. Barr, William J. Panenka, Caroline A. MacCallum

**Affiliations:** ^1^Department of Medicine, Faculty of Medicine, University of British Columbia, Vancouver, BC, Canada; ^2^Department of Psychology, Queens University, Kingston, ON, Canada; ^3^Centre for Neuroscience Studies, Queens University, Kingston, ON, Canada; ^4^Department of Emergency Medicine, Faculty of Medicine, University of British Columbia, Vancouver, BC, Canada; ^5^Department of Anesthesiology, Pharmacology & Therapeutics, Faculty of Medicine, University of British Columbia, Vancouver, BC, Canada; ^6^British Columbia Mental Health and Substance Use Services Research Institute, Vancouver, BC, Canada; ^7^Department of Psychiatry, Faculty of Medicine, University of British Columbia, Vancouver, BC, Canada; ^8^British Columbia Provincial Neuropsychiatry Program, Vancouver, BC, Canada

**Keywords:** cannabinoids, medical cannabis, tetrahydrocannabinol, cannabidiol, pain, impairment, intoxication, cognition

## Abstract

While the recreational use of cannabis has well-established dose-dependent effects on neurocognitive and psychomotor functioning, there is little consensus on the degree and duration of impairment typically seen with medical marijuana use. Compared to recreational cannabis users, medical cannabis patients have distinct characteristics that may modify the presence and extent of impairment. The goal of this review was to determine the duration of acute neurocognitive impairment associated with medical cannabis use, and to identify differences between medical cannabis patients and recreational users. These findings are used to gain insight on how medical professionals can best advise medical cannabis patients with regards to automobile driving or safety-sensitive tasks at work. A systematic electronic search for English language randomized controlled trials (RCTs), clinical trials and systematic reviews (in order to capture any potentially missed RCTs) between 2000 and 2019 was conducted through Ovid MEDLINE and EMBASE electronic databases using MeSH terms. Articles were limited to medical cannabis patients using cannabis for chronic non-cancer pain or spasticity. After screening titles and abstracts, 37 relevant studies were subjected to full-text review. Overall, seven controlled trials met the inclusion/exclusion criteria and were included in the qualitative synthesis: six RCTs and one observational clinical trial. Neurocognitive testing varied significantly between all studies, including the specific tests administered and the timing of assessments post-cannabis consumption. In general, cognitive performance declined mostly in a THC dose-dependent manner, with steady resolution of impairment in the hours following THC administration. Doses of THC were lower than those typically reported in recreational cannabis studies. In all the studies, there was no difference between any of the THC groups and placebo on any neurocognitive measure after 4 h of recovery. Variability in the dose-dependent relationship raises the consideration that there are other important factors contributing to the duration of neurocognitive impairment besides the dose of THC ingested. These modifiable and non-modifiable factors are individually discussed.

## Introduction

The legalization and decriminalization of cannabis in multiple countries and states has contributed to a wealth of research on the potential therapeutic benefits of cannabis-based medicines (1–5). In 2014, cannabinoids were deemed appropriate as third-line treatment for neuropathic pain by the Canadian Pain Society (6). Cannabis has also been investigated as an adjuvant in refractory chronic non-cancer pain and in harm-reduction approaches for those tapering off high-dose opioid medications, with promising preliminary findings (7–11). As the indications for cannabis expand beyond neuropathic pain, seizures and multiple sclerosis (MS)-related spasticity, it is necessary to assess the risks associated with medicinal cannabis use, especially among those who regularly ingest THC-containing compounds.

Research on the effects of cannabis on humans has largely focused on recreational use, with smoking as the most common route of administration. This early work found strong associations between the dose of THC inhaled and resulting acute cognitive impairment (12). Specifically, THC and other cannabinoid receptor 1 (CB_1_) agonists acutely impair psychomotor and neurocognitive domains including attention, manual dexterity, coordination, and reaction time, as CB_1_ receptors are neuroanatomically expressed in regions responsible for cognitive and motor control (13, 14). Therefore, THC dose-dependently disrupts important cognitive and psychomotor functions needed for safety-sensitive work, including driving motorized vehicles (15, 16).

There is currently no standardized definition of impairment associated with medical cannabis use in the literature and therefore, no general consensus on how to measure or define this impairment. Unlike with alcohol, where blood alcohol levels directly correlate with the degree of intoxication, the relationship between cannabinoid and neurocognitive or functional impairment remains undetermined. While evidence supports a positive relationship between THC dose and impairment, an accurate blood concentration range has not been determined (17). Some studies have suggested THC blood concentrations between 2 and 5 ng/ml are associated with impairment (18–20). However, these measures do not consistently correlate with impairment across individuals (17, 21). This is likely due to the complex nature of THC pharmacokinetics and metabolism (17, 20) which is strongly impacted by individual factors such as genetics and tolerance to THC.

The two main metabolites of THC include the primary psychoactive metabolite “11-hydroxytetrahydrocannabinol” (11-OH-THC) and the second metabolite “11-nor-9-carboxy-tetrahydrocannabinol” (THC-COOH) (22). The latter is a non-psychoactive and non-intoxicating cannabis metabolite which is usually eliminated from the body within 5 days of consumption primarily via feces and urine (23). From recreational cannabis studies, the detectable half-life of THC-COOH is much longer than for THC and 11-OH-THC. For infrequent cannabis users the half-life of THC-COOH is around 1.3 days, while for frequent users it is in the range of 5–13 days (24). The practical implication for medical cannabis patients is that they would likely test positive for cannabis on urine drug tests (which typically detect THC-COOH) days after last using THC (22). As THC-COOH is not psychoactive, its prolonged presence in frequent users is not a valid biomarker of impairment.

There is evidence that medical cannabis patients who use THC regularly develop tolerance to many of the impairing effects of THC (25). Tolerance has also been found with recreational cannabis use, with experimental studies demonstrating that frequent recreational cannabis users, with use more than four times per week, developed psychological and behavioral tolerance, and showed no significant impairment in neurocognitive function or motor side effects compared to infrequent users at the same dose of THC (26, 27). Other research demonstrates that tolerance is incomplete, and people who use cannabis regularly still demonstrate some impairment, albeit blunted, after acute use (28).

Determining the duration of potential THC impairment, and what THC dose a medical cannabis patient should take to minimize neurocognitive impairment, proves to be challenging. There are some unique considerations when studying impairment in medical cannabis patients, defined here as someone who uses cannabis under the guidance of a medical practitioner, compared to recreational cannabis users. Medical cannabis patients often use THC to manage symptoms for a variety of conditions including chronic pain, insomnia, PTSD, autoimmune conditions, and neurological disorders, that induce a certain level of neurocognitive impairment by themselves. By treating these symptoms, their neurocognitive and psychomotor functioning may actually improve. Medical cannabis patients also have different patterns of use, including a more consistent and standardized dosing schedule, along with different expectations and goals (29). They often consume cannabis orally, which lengthens the time until onset and the duration of effect after use, and choose use chemovars high in cannabidiol (CBD), which is non-impairing (30). If medical cannabis patients are starting THC, most start with low-dose THC products, with doses titrated to obtain symptomatic relief while purposely avoiding impairing side-effects.

The aim of the present scoping review was to identify and summarize studies that investigate the duration and degree of acute neurocognitive impairment with medical cannabis use, and to compare this literature with the body of research on neurocognitive impairment in recreational cannabis users (31–35). Impairment, for the purposes of this review, is considered as disruption in neurocognitive and motor tasks that, if present, could potentially cause harm to the subject or others (e.g., driving or workplace safety). To investigate this critical question, we performed a scoping review of clinical trials that used standardized neurocognitive and psychomotor tests to study medical cannabis patients preceding and following acute THC administration. These findings are then compared to similar research involving recreational cannabis users to explore unique features of the medical cannabis patient population. We conclude by proposing a provisional standardized neurocognitive and psychomotor assessment battery for studying acute THC impairment in medical cannabis patients, and by discussing how medical professionals can best advise patients with regards to safety-sensitive work, including driving.

## Materials and Methods

This study is a scoping review and qualitative analysis of the literature on impairment in medical cannabis patients. A systematic electronic search for English language randomized controlled trials (RCTs), clinical trials and systematic reviews (in order to capture any potentially missed RCTs) between 2000 and 2019 was conducted through Ovid MEDLINE and EMBASE electronic databases using the following MeSH terms: (exp Cannabinoids/ OR cannabi^*^ OR dronabinol OR marijuana OR tetrahydrocannabinol OR THC OR Sativex) AND (chronic non? cancer pain OR Chronic Pain/OR muscle spasticity/OR spasticity) AND (impair^*^ OR cognition OR intoxication OR reaction time OR coordination OR neurocognitive OR psychomotor). This search strategy was developed with the assistance of a medical librarian, and was conducted as we have previously reported on prior studies of drug-associated psychological effects (36–38).

Titles and abstracts were reviewed and obviously irrelevant studies were excluded. Full text of the remaining studies was reviewed to determine eligibility. The review was performed by a single investigator. Input from a second investigator was sought as required. The current focus was on medical cannabis patients using cannabis for chronic non-cancer pain or spasticity. Studies were included if they documented dose, product type and method of THC administration in addition to having formal objective neurocognitive or psychomotor baseline and acute post-THC assessments. See Table 1 for PICO statement. Abstracts were analyzed for inclusion based on PRISMA criteria. Studies were excluded if they focused solely on recreational cannabis use, did not have any objective neurocognitive or psychomotor testing, or if the testing was done following subacute exposure, such as after weeks or months of daily THC exposure (Table 2).

**Table 1:** PICOS breakdown of study eligibility criteria.

P (Problem or Patient or Population)	Adults living with chronic, non-cancer pain (pain of >3-month duration) and/or spasticity.
I (Intervention/indicator)	Medical cannabis use or cannabinoid-based medicines.
C (Comparison)	Chronic pain/spasticity controls (without cannabis use). Studies without comparators will also be included.
O (Outcome of interest)	Duration of acute neurocognitive and psychomotor impairment using objective standardized measures
S (Study types selected)	Randomized controlled trials and other clinical trials will be included.

**Table 2:** Inclusion and exclusion criteria (for medical cannabis patients using cannabis for chronic non-cancer pain or spasticity).

**Inclusion criteria**	
	Cannabis and the management of chronic non-cancer pain and/or spasticity
	Efficacy, tolerability, and safety studies on the use of medical cannabis for chronic non-cancer pain and/or spasticity
**Exclusion criteria**	
	Studies in a language other than English
	Studies published before 2000
	Studies which focus on recreational cannabis use
	Studies focusing on cannabis use disorder
	Studies without any formal and objective/reproducible neurocognitive testing
	Studies investigating the non-acute use of cannabis (for example, impairment after using daily THC for 1 month, instead of 1 h-post consumption)
	Studies on animals

Systematic reviews on medical cannabis use were also evaluated. Three additional RCTs that met the inclusion criteria were found in the references of these systematic reviews and were added to the analysis. One newly published observational clinical trial discovered through exert recommendation was added to the final analysis that was not found in our original electronic search. A database was not created from our review.

Data extracted from the investigated studies included the type of study completed, the number of participants, the participant characteristics, such as their medical condition causing pain or spasticity and their previous experience with cannabis, (or presumed THC tolerance), the THC concentrations assessed, the THC dosing intervals, the neurocognitive tests utilized, the timing of the neurocognitive testing intervals and the results of these neurocognitive tests for each THC dose and timing interval. The data drawn from the included studies was interpreted and summarized to make a preliminary recommendation on the duration of neurocognitive and motor impairments in medical cannabis users.

## Results

**Figure 1:**
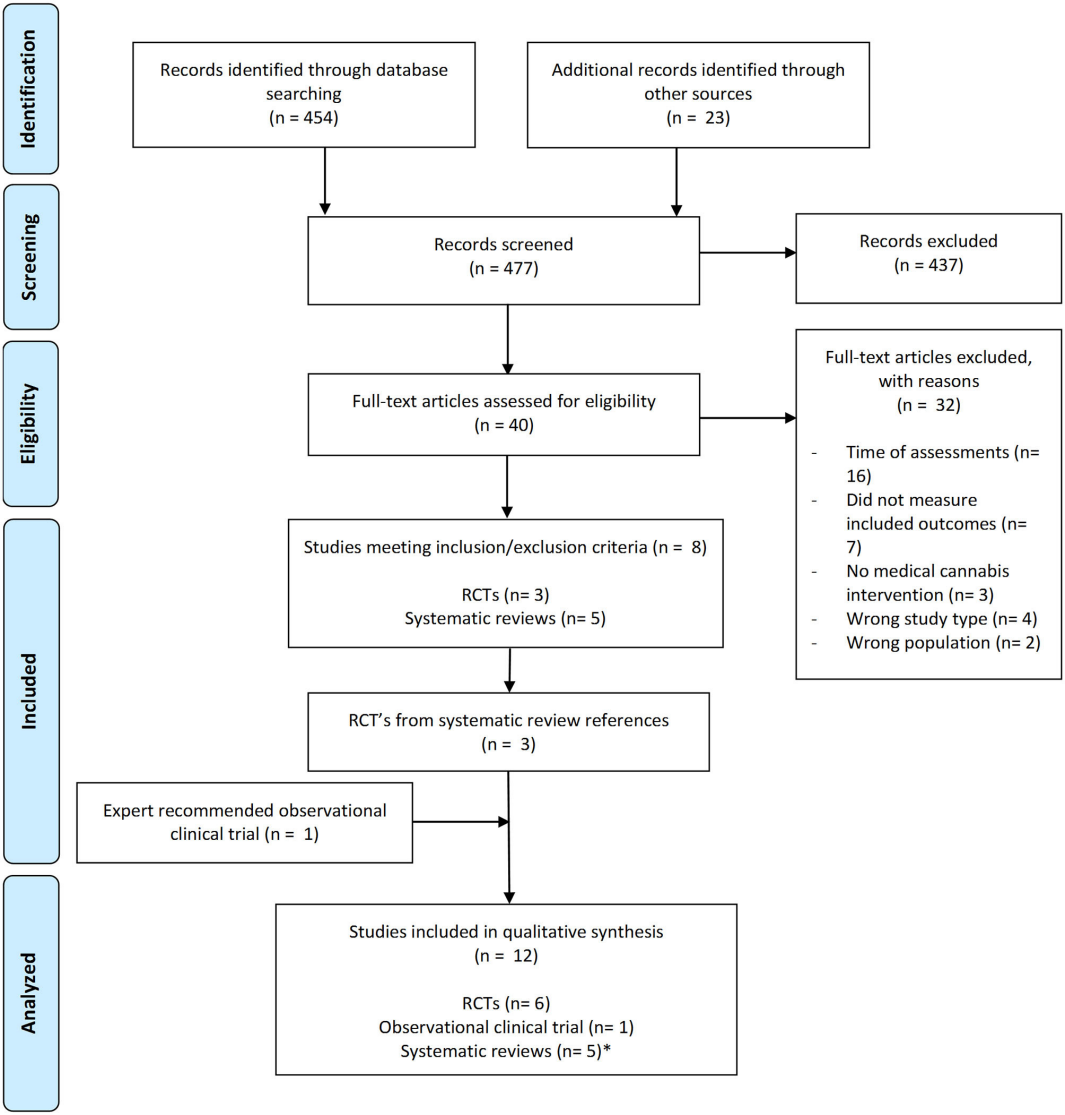
Flow diagram of search strategy and methodology. * Results from systematic reviews were not included in our formal analysis as we were comparing raw data from independent clinical trials.

We identified 454 potentially eligible publications from the search strategy and twenty other potential articles from other resources. After screening titles and abstracts, 37 relevant studies were subjected to full-text review. One review article analyzed contained three additional RCTs which were independently reviewed for a total of 40 relevant studies reviewed. 32 studies were excluded for the following reasons: they measured subacute impairment of THC (days to weeks after ingestion), they did not have formal neurocognitive testing, there was no formal medical THC intervention completed, the study was not interventional, or they did not study adults living with chronic, non-cancer pain and/or spasticity. Eight studies met our final criteria, five systematic reviews and three RCT's. From the systematic reviews, three RCT's were extracted for analysis. One newly published observational clinical trial discovered through expert recommendation was added that was not found in our original search. Overall, seven controlled trials met the inclusion/exclusion criteria and were included in the qualitative synthesis: six RCTs and one observational clinical trial. A flow diagram of our search strategy summarizes our methodology (Figure 1).

### Study Characteristics

Study characteristics for the six RCTs and one observational trial are summarized in Table 3 (39–45). A total of 234 medical cannabis patients were included in these studies: 175 patients with neuropathic pain, 37 patients with MS-associated spasticity and 22 patients prescribed medical cannabis pre-dominantly for chronic pain, anxiety or depression.

**Table 3:** Study characteristics and results.

**Study**	**Population**	**Intervention**	**Cannabis use**	**Outcome**	**Results**
Wallace et al. (39) Randomized, double-blind, placebo-controlled crossover study	Painful Diabetic Neuropathy 16 participants	Placebo, 1, 4, and 7% THC vaporized 4 inhalations using the Foltin Puff Procedure in one single dosing session (equaling 0, 4, 16, or 28 mg THC)	No use of cannabis in past 30 days prior to study tested by urine drug screen	Trail Making Test Paced Auditory Serial Attention Test Testing at 5-min, 30-min and every 30- min for 3 h. Final measurement at 240-min.	Decline in neurocognitive performance with THC exposure which was dose dependent and improved with time. No difference in any groups at 240-min post-inhalation (4-h). *Trails*: 7% THC group took longer compared to placebo on Trails B at 120-min. No difference between 1 and 4% THC groups and placebo *Paced Auditory Serial Addition Test*: 7% THC and 4% THC groups had worse performance than placebo at 15-min post-THC dose. There was no difference in performance between 1, 4, or 7% THC groups compared to placebo at the following 60-, 120-, or 240-min testing.
Wilsey et al. (40) Double-blind, placebo-controlled, crossover study	Central and Peripheral Neuropathic Pain 38 participants	Placebo vs. 3.5% THC vs. 7% THC smoked 2 inhalations at 60-min, 3 inhalations at 120-min, and 4 inhalations at 180-min for a total of 9 cumulative inhalations (total estimate: 19 mg THC low dose, 34 mg THC high dose)	All had previous cannabis exposure No cannabis 30 days prior to study	Digit Symbol Test Hopkins Verbal Learning Test and Delayed Learning Grooved Pegboard Dominant and Non-Dominant tests Testing completed at baseline, 60-mins (after 2 puffs), 120-min (after 3 puffs), 180-mins (after 4 puffs), 240-min (after 1-h recovery).	Modest decline in cognitive performance with THC use, most significant in the 7% THC group. 76% of participants had cognitive impairment at baseline. *Digit Symbol Test:* no significant dose-effect differences *Hopkins:* 7% THC group had worse performed than the 3.5% THC group which performed worse than placebo. Poor performance even in placebo group *Dominant-hand Pegboard*: 7% THC group performed worse than placebo. No difference in performance between the 3.5% THC group and placebo. *Non-dominant hand pegboard:* Both THC groups had decreased performance compared to placebo. 2-h after the last inhalation session, both THC groups had significant improvement compared to their previous scores
Corey-Bloom et al. (41) Randomized placebo-controlled trial	Multiple Sclerosis Spasticity 37 participants	Placebo vs. 4% THC smoked 4 inhalations of 4% THC smoked in one dosing session (~16 mg THC)	Cannabis naïve or negative toxicological screen for THC at study initiation	Timed walk score Paced Auditory Serial Addition Test Baseline and 45-min post-treatment	*Timed walk*: no difference *Paced Auditory Serial Addition Test*: 4% THC group had worse performance compared to placebo at 45-min. There was no neurocognitive testing beyond 45-min.
Notcutt et al. (42) Prospective, randomized, double-blind, placebo-controlled crossover study	Chronic mostly neuropathic pain 34 participants	Sublingual Spray 2.5 mg THC vs. 2.5 mg CBD vs. 2.5 mg THC and 2.5 mg CBD One spray every 15–30 min and individually stopped further dosing after response was achieved Total intake: 2–8 sprays over a 4-h period (~5–20 mg THC)	Excluded if significant past or current recreational cannabis use, okay if medical cannabis use	Trail Making Tests A & B Adult Memory and Information Processing Battery Baseline and 3-h post-dose	Equivocal results, requiring a more detailed analysis than the study planned. Testing often improved after the initiation of cannabis-based medicine.
Wilsey et al. (43) Crossover, randomized, placebo-controlled human laboratory experiment	Patients with refractory neuropathic pain who have disease or injury to their spinal cord 48 participants	Placebo vs. 2.9% vs. 6.7% THC vaporized 4 puffs using the Foltin Puff Procedure at 60-min with a second dosing session at 240-min of 4–8 puffs (flexible dosing schedule: the participant chooses their second dose between 4–8 puffs)	17/42 participants used cannabis regularly Some were cannabis naïve or ex-users	Wechsler Adult Intelligence Scale Digit Symbol Test Trail Making Test Grooved Pegboard Test Paced Auditory Serial Addition Test Hopkins Verbal Learning Test Revised with 20-min delay Neurocognitive testing every hour (with variations to prevent learning)	Measurement of neurocognitive performance proved technically challenging due to the various disabilities in the population studied. THC showed dose-dependent neurocognitive impairment with resolution 2 h after inhalation of THC. *Dominant-hand Pegboard:* no significant dose-effect differences *Non-Dominant Hand Pegboard*: 6.7% THC group performed worse compared to placebo 1-h after the 2nd THC dosing session. Resolved 1-h later *Digit Symbol Test*: no significant dose-effect differences, with all groups improving scores over time, consistent with practice effects *Trail Making Test-A*: 2.9% THC group took longer than the 6.7% THC group on the Trails A at 420 min, immediately after the 2nd THC dosing interval *Hopkins*: no difference in test scores between the 2.9% THC group and placebo. 6.7% THC group had less true-positive and more false-positive responses compared to placebo. Resolved 2-h after the 2nd dosing session *Paced Auditory Serial Addition Test*: no significant differences between THC groups and placebo at any timepoint. 6.7% THC group performed better than the 2.9% THC group at 420 min, 3-h after the 2nd dosing interval
Wilsey et al. (44) Randomized double-blind placebo controlled cross-over trial	Central or peripheral neuropathic pain (Refractory) 39 participants	Placebo vs. 1.29%, vs. 3.53% THC vaporized 4 puffs at using the Foltin Puff Procedure at 60-mins with a second dosing session at 180-min of 4–8 more puffs (flexible dosing schedule: the participant chooses their second dose between 4 and 8 puffs)	All had previous cannabis exposure No cannabis 30 days prior to study	Wechsler Adult Intelligence Scale Digit Symbol Test Hopkins Verbal Learning Test Revised Grooved Pegboard Test Baseline, 60-, 120-, 180-, 240-, and 300-min after administration of THC	THC produced a short duration of neurocognitive impairment. No difference in performance between THC and placebo 2-h after the last dosing session *Digit Symbol Test*: 1.29 and 3.53% THC groups had worse performance at 60-min, (after 1st inhalation) and 180-min, (after the 2nd inhalation) compared to placebo. No difference in either THC group and placebo at 120- and 240-min (1-h after each dose) *Dominant Hand Pegboard*: 1.29% THC group had worse performance than the 3.53% THC and the placebo group at 60-min (after 1st inhalation) and 240-min, (60-min after 2nd inhalation) which resolved 60-min later *Non-dominant Hand Pegboard*: 1.29% THC and 3.53% THC groups had decreased performance at 120-min (60-min after 1st inhalation) and 180-min (after 2nd inhalation) which resolved 60-min later *Hopkins*: performance following higher THC doses was worse than for lower doses of THC, which in turn, were worse than placebo. There was recovery of these differences 2-h after the last THC inhalation session.
Olla et al. (45) Observational Clinical Trial	Medical Cannabis Patients 22 participants	One gram 20% THC in vapes, cannabis cigarettes (joints) and dabs for 10 min One dosing session with 10 min of THC intake	Regular cannabis use ≥6 month 3.2 g/day cannabis average)	Brief Neurocognitive Battery: Animal Fluency, Boston Naming Test-15, Coding, Digit Span, Stroop Color Naming/Word Reading/Interference, Trails Making Test A/B Baseline, 30 min and 2.5–3 h after intake Included Performance Validity Testing	There was no psychometric evidence for a decline in performance on cognitive testing following THC ingestion and some participants had improved performance after THC ingestion compared to the normative sample. Performance Validity Test: More failures in the THC group, which were the most affected parameters of the suppressing effects of THC on cognitive functioning.

Route of cannabis administration varied: two studies required patients to smoke cannabis, three used vaporized cannabis, one allowed for smoking or vaporizing, and one study used sublingual THC, CBD, or THC: CBD spray. All three vaporization studies utilized the Foltin Puff Procedure, where participants are verbally signaled to “hold the vaporizer bag with one hand and put the vaporizer bag mouthpiece in their mouth” (30 s), “get ready” (5 s), “inhale” (5 s), “hold vapor in lungs” (10 s), “exhale and wait” before repeating the puff cycle (40 s) (39, 43, 44).

Four of the seven studies required participants to abstain from non-study cannabis use for at least 30 days prior to the start of the study (39, 40, 44). Two of the four verified abstinence through negative urine drug screens (39, 41). Several of the studies allowed medical cannabis use prior to the study initiation (42, 45), with less than half of the participants from one study reporting regular cannabis use (43).

There were a variety of testing protocols, with significant variability on the timing of THC or placebo administration and when the neurocognitive testing was completed. Some studies performed a single THC administration (39, 41, 45), where others had cumulative inhalation procedures (40, 42–44). Neurocognitive testing was either singular or repeated, with the most complete testing at baseline and every 30 min for 3 h total after THC ingestion (39).

### Summary of Findings

Neurocognitive testing varied significantly between all studies, including the specific tests administered and the timing of assessments post-cannabis consumption. Table 3 provides findings from individual studies, while Table 4 provides details about the neurocognitive tests administered and the cognitive modalities examined with each test.

**Table 4:** Neurocognitive tests and cognitive domains.

**Neurocognitive test**	**Neurocognitive correlate assessed**
Paced Auditory Serial Attention Test	Auditory information processing speed and working memory
Wechsler Adult Intelligence Scale Digit Symbol Test	Concentration, psychomotor speed, and graphomotor abilities
Trail Making Test A and B	Processing speed, visual attention, and task-switching
Grooved Pegboard Test (Dominant and Non-Dominant)	Fine motor coordination and speed
Hopkins Verbal Learning Test Revised with 20-min delay	Learning/ability to retain, reproduce, and recognize information after a 20 min delay. Immediate and delayed recall of verbal information
Adult Memory and Information Processing Battery	*Spatial Recall Test*: Visuospatial memory *Symbol Digit Modalities Test*: Concentration, psychomotor speed, and graphomotor abilities *Paced Auditory Serial Addition Test*: Auditory information processing speed and working memory *Word Generation List*: Lexical fluency *Selective Reminding Test*: Verbal learning and memory
Brief Neurocognitive Battery	*Animal Fluency*: Semantic fluency and executive control *Boston Naming Test-15*: Expressive language *Coding*: Attention and visuomotor processing *Digit Span*: Auditory attention and working memory *Stroop Color Naming*: Attention and speed of information processing *Stroop Word Reading*: Attention and speed of word reading *Stroop Interference*: Inhibition and cognitive flexibility *Trails Making Test-A*: Simple attention, visual scanning and processing speed *Trails Making Test-B*: Visual scanning, divided attention and cognitive flexibility

Two of the three studies using the Trails Making Test to assess visual attention and processing speed with switching tasks did not find significant differences between THC groups compared to placebo except for at two timepoints (39, 43). In one study, the low-dose THC group took longer than the high-dose THC group on the Trails A at 420 min, immediately after the second THC dosing interval (43). The second study found the high dose group took longer compared to placebo on the Trails B at 120 min post-dose (39). The third study assessing the Trails Making Test did not report their quantitative results in their findings (42).

Of the three studies using the Paced Auditory Serial Attention Test for auditory processing speed and working memory (39, 41, 43), one study found no significant differences between THC groups and placebo at any timepoint, but the high-dose THC group performed better than the low-dose THC group at 420 min (43). In the second study, the high- and medium-dose THC groups had worse performance than placebo at 15 min post-inhalation, but there was no difference in performance between low, medium, or high dose THC groups compared to placebo at the following 60-, 120- or 240-min post-inhalation testing (39). In the final study, the THC group had worse performance compared to placebo at 45 min post-inhalation with no further testing after this timepoint (41).

Results were mixed between the three studies using the Grooved Pegboard Test (GPT) (40, 43, 44) to assess dexterity and fine motor control. All three studies used cumulative cannabis inhalation protocols. One study found no significant effects across active doses compared to placebo on the dominant-hand GPT but observed decreased performance on the non-dominant GPT in the high-dose THC group compared to placebo. This occurred 1-h after the second THC dosing session and resolved after an additional 60 min (43). In the second study, the low-dose THC group had worse performance than the medium-dose THC and the placebo group on the dominant-hand GPT at 60 min, (immediately after the first dosing session), and 240 min, (60 min after the second dosing session) (44). This same study found that both the low-dose and medium-dose THC groups had decreased performance on the non-dominant GPT at the 120- and 180-min (60 min after first dosing session and immediately after the second dosing session) (44). There was no difference in performance between placebo and either THC group at the 300-min mark, 3 h after the last scheduled inhalation (44). The final study found a decrease in overall performance in the high-dose THC group compared to placebo on the dominant-hand GPT, but no difference between the low-dose THC group and placebo. In the non-dominant hand GPT, this study found that both THC groups had decreased performance compared to placebo. The study measured maximal recovery 2 h after the last inhalation session at 180 min where low-dose and high-dose THC groups had significant improvement on the GPT compared to their previous scores (40).

All three studies that administered the Hopkins Verbal Learning Test and Delayed Learning Test to assess learning, immediate and delayed recall found THC dose-dependent impairment on learning and recall compared to placebo (40, 43, 44). For two studies, performance following higher THC doses was worse than for lower doses of THC, which in turn, were worse than placebo (40, 44). Notably, one study found poor performance on this test even in the placebo group, hypothesized to be due to their underlying neuropathic pain condition (40). The second study found recovery of these differences 2 h after the last inhaled THC session (44). The final study found no difference in test scores between the low-dose THC group and placebo. In this study, the high-dose THC group had fewer true-positive responses and more false positives compared to placebo, a difference that resolved 2 h after the second dosing session (43).

Two of the three studies administering the Wechsler Adult Intelligence Scale Digit Symbol Test to assess concentration and graphomotor speed found no significant dose-effect differences throughout the duration of the study (40, 43) with one study noting improvement among all conditions (including placebo), consistent with a learning effect (43). The remaining study found a decrease in performance at 60-min, (immediately after first inhalation session), and 180-min, (immediately after the second inhalation session), in both the low dose and high dose THC groups compared to placebo, although there was no difference between placebo and either THC group 1 h after each dosing session (44).

One study used the Adult Memory and Information Processing Battery in addition to Trails Making Test, although the authors did not report their results (42). The final study utilized the Brief Neurocognitive Battery (Table 4), consisting of a comprehensive series of neurocognitive tests with combined Performance Validity Testing-additional tests that are robust to the effect of genuine impairment and allow for the determination of the impact of the patient's effort or engagement in testing (45). Cannabis patients were compared to the normative sample supplied with the Brief Neurocognitive Battery technical manual and were also compared to test results from 40 non-cannabis using Canadian UG students completing this test battery unimpaired. Medical cannabis patients either matched or outperformed both the normative data set and the Canadian UG students test results at 30 min and 150–180 min post-THC ingestion, showing no evidence of neurocognitive impairment following THC consumption. (45).

In summary, there is evidence that cognitive performance declined mostly in a THC dose-dependently manner, with steady resolution of impairment in the hours following THC administration. There is some variability in this dose-dependent relationship, bringing forward the consideration that there are other important factors contributing to the duration of neurocognitive impairment besides the dose of THC ingested. For example, one study found no neurocognitive impairment, and even higher neurocognitive test scores in the THC group compared to the normative data set (42, 43, 45). In all the studies, there was no difference between any of the THC groups and placebo on any neurocognitive measure after 4 h of recovery (39).

## Discussion

This scoping review provides evidence that cognitive performance in medical cannabis patients acutely declines after THC use, with steady resolution of impairment in the hours following THC administration. The degree of impairment is predominantly dose-dependent; higher doses of THC are generally more impairing than the lower doses. The duration of neurocognitive impairment varied between studies, partly due the heterogeneity in study designs. Nonetheless, there was no difference on any neurocognitive test between placebo and the active THC groups at 4-h of recovery, irrespective of the THC dose inhaled (39–45). Importantly, none of the studies collected blood to measure plasma levels of THC and its metabolites. It would have been informative to have been able to directly relate objectively measured cognitive impairment across specific domains to plasma levels of cannabinoids in these subjects.

**Figure 2:**
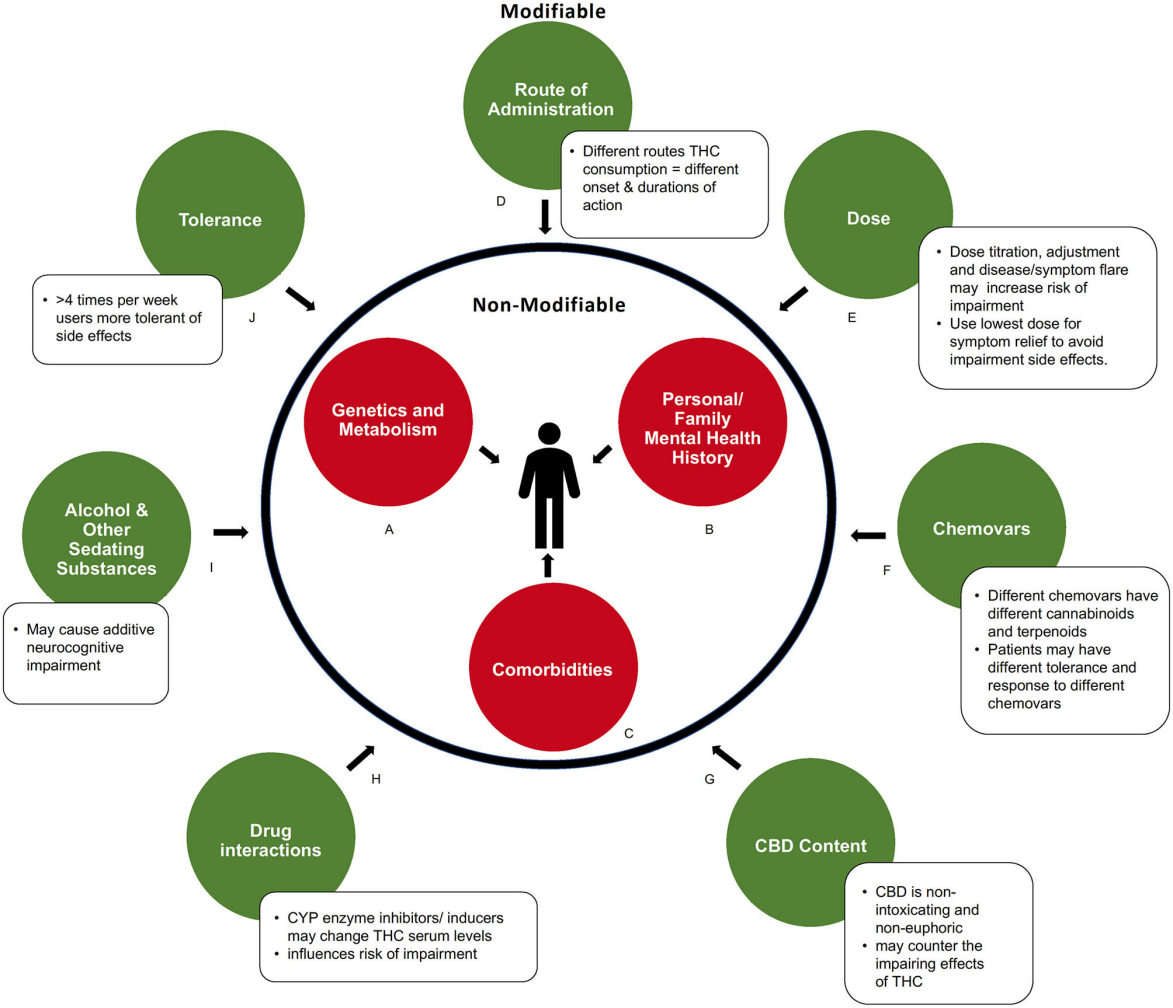
Modifiable and non-modifiable factors influencing acute neurocognitive impairment in medical cannabis users. **(A)** Genetic and metabolic profiles can influence response to cannabinoids. **(B)** Predisposition to or history of mental health conditions may increase risk of impairment. **(C)** Comorbidities that produce symptoms like fatigue, dizziness, or cognitive slowing may compound impairment. **(D)** How cannabis is consumed influences the duration of impairments via differences in absorption and metabolism. **(E)** Severity of impairment is THC dose-dependent. **(F)** Chemical composition (level of various cannabinoids and metabolites) of a cannabis product influences degree of impairment **(G)** Amount of CBD contained in product may balance side effects of THC. **(H)** Drug interactions can alter serum THC levels. **(I)** Use of other sedating recreational or prescribed substances may cause additive impairment. **(J)** Pattern of regular consumption in medical cannabis users decreases drug response, and side effects, to cannabinoids.

Several observations from this review draw important comparisons with the recreational cannabis literature. As we have already discussed in detail the results of the scoping review and the seven studies in the Summary of Findings above, the focus on the present Discussion is to highlight and discuss important considerations when reviewing the current literature in addition to a variety of modifiable and non-modifiable factors that were found to influence the duration and degree of neurocognitive impairment in medical cannabis patients (see Figure 2).

There are several non-modifiable factors, intrinsic to the patient, that influence both the degree and duration of impairment (Figures 2A–C). These important factors are sometimes overlooked within the larger body of literature, particularly within recreational studies.

### Genetics and Metabolism

Genetic and metabolic profiles or predispositions influence how an individual responds to cannabis, and thus the side effects experienced. Genetics, such as variations in the COMT/AKT genotype (46, 47), individual endocannabinoid system “tone” [endogenous endocannabinoid levels, receptor sensitivity and abundance, which may be altered in psychiatric conditions such as depression (48, 49)], as well as hypo- or hypermetabolizers can influence how THC is metabolized (50) and thus the degree and duration of impairment experienced by an individual (Figure 2B). This may influence study outcomes, particularly when smaller sample sizes are used.

### Personal or Family Mental Health History

It is important to consider personal or family mental health history when assessing factors of impairment. Experienced or known pre-dispositions to some mental health conditions may increase the risk of impairment for some individuals (Figure 2B) (51, 52). The use of high THC chemovars may exacerbate this risk.

### Comorbidities

Studies that assess the therapeutic effects of THC based on ability to manage symptoms, predominantly pain or spasticity, should acknowledge that these symptoms may contribute to impairment (Figure 2C). Patients with certain medical conditions, such as multiple sclerosis, epilepsy, insomnia, anxiety, and depression, have twice the risk of motor vehicle accidents than healthy controls (53–55). Chronic pain syndromes can manifest with comorbid fatigue, weakness, dizziness, or cognitive slowing, which may compound the impairment produced by THC. However, by managing these symptoms with medical cannabis, baseline neurocognitive and psychomotor functioning may improve, as was reported in a driving simulation study with patients who have multiple sclerosis (56). Co-morbidities with additive impairing effects should be carefully considered clinically and in future research. In addition to non-modifiable factors, this review identified several modifiable factors that were found to influence the duration and degree of impairment. These are now discussed in more detail below (Figures 2D–K).

### Route of Administration

As represented in Figure 2D, there is a clear difference in the duration of neurocognitive impairment depending on the route of administration (smoked vs. sublingual spray vs. oils). Due to differences in absorption and metabolism, THC has a different onset and duration of action depending on where in the body it is administered (57–59). Cannabis oils may provide up to 8 h of symptom relief due to gradual absorption of THC from the gut combined with first pass metabolism conversion of THC to 11-OH-THC, another active compound, in the liver (30, 58). The longer duration of therapeutic action also gives ingested formulations a greater period of potential impairment. Inhaled or vaporized THC produces a shorter period of impairment compared to oral formulations, with typical onset with 5–10 min and duration for 3–4 h. This is due to rapid absorption of THC from the lungs into the bloodstream, with minimal conversion to 11-OH-THC by the liver via first-pass metabolism (30, 60–62). Although none of the studies above utilized oil ingestible THC formulations, clinically this is a common method of intake for patients using medical cannabis, to limit the negative effects of smoking. We would recommend that future studies administer cannabis oils, providing doses similar to those that are prescribed in practice, in order to appropriately represent the medical cannabis population. Further, new formulations are being manufactured with different carrier oils, extraction techniques, and cannabinoid content which may lead to different levels and duration of impairment. Future pharmacokinetic studies assessing these formulations are needed.

### Dose

The degree and duration of neurocognitive impairment is dose-dependent, with higher THC doses being more impairing than lower doses. The dose of THC used among the medical cannabis studies reviewed were substantially lower compared to typical recreational studies (Figure 2E). Recreational studies often measure neurocognitive functioning in heavy cannabis users and follow the participants usual cannabis regimen, with a reported average of two cannabis “joints” per dosing session (63–66). If one “joint” contains ~750 mg of cannabis with a THC concentration of 15%, one dosing session would contain 225 mg of THC. Some of these high-dose THC recreational studies have shown subtle defects in cognitive tasks up to 24-h after THC inhalation (65). However, recreational studies using doses similar to this medical cannabis review, [with the highest dose administered being 34 mg of THC (40)], do not note any neurocognitive impairment 24-h after THC ingestion (67).

Rather than using data from studies with medical cannabis users and with doses typically used by medical cannabis patients, Health Canada's “Cannabis Impairment” report based its conclusions on data from studies of recreational cannabis, where doses are substantially higher. The report notes: “(s)ome effects of cannabis use, for example drowsiness, can last up to 24 h, well after other effects may have faded…(T)here is no standard waiting time to drive after using cannabis. If you are using cannabis, do not drive.” (68). If they followed these recommendations, many daily medical cannabis patients would be unable to drive or attend work, even if they only utilize THC at night before going to sleep.

This review of the literature found no reports of neurocognitive deficits with THC use 4-h after inhalation using modest THC-dosing strategies. We would recommend using lower-THC doses, (as were seen in the studies in this review), for daily symptom management, as higher doses may prolong the duration of impairment.

### Chemovars and CBD Content

The addition of other cannabinoids, such as CBD, may have an impact on the severity of neurocognitive impairment (Figures 2F,G) (69). One of the studies in this review, compared oromucosal spray formulations of THC vs. THC: CBD 1:1 vs. CBD vs. placebo and noted that participants in the THC: CBD group had less drowsiness, dysphoria, and euphoria (Figure 2F) (42). In addition to CBD, cannabis contains many other cannabinoids and terpenes that may affect neurocognitive impairment (Figure 2F). For example, myrcene may potentiate the sedating effects of THC (70, 71). Importantly, this could mean that patients who develop tolerance to the unwanted neurocognitive side effects of one chemovar of cannabis may not have the same tolerance to other chemovars with different concentrations of cannabinoids and terpenoids (70). Thus, another informative avenue for future studies would be to monitor and record in detail the quantities and concentrations of the other constituents of the cannabis being studied, as the individual or “entourage” effects of these on cognitive impairment is largely unknown.

### Drug Interactions and Sedating Substances

Medical cannabis patients often utilize other impairing substances to manage their conditions. The interaction of these substances with THC may further the duration and severity of neurocognitive impairment (Figures 2H,I). For example, there is the potential for additive impairment due to interactions with other intoxicants (e.g., alcohol) or sedating medications such as benzodiazepines, opioids, tricyclic antidepressants, and anti-epileptics (Figure 2I) (58). All studies in the current review required patients to stay on their normal routine medications (39–45), and only one study excluded participants who were on opioid medications or used any other medication deemed to interact with cannabis (45). The articles in this review did not list which medications were routinely consumed by patients, which would have been useful information. Most of the articles provide a brief summary of the major medical conditions that were associated with medical cannabis use, so some inferences can be drawn, but detailed information is missing. In clinical practice, it has been commonly noted that many patients reduce their use of prescription medications if they achieve greater symptom relief with marijuana, which can actually reduce overall sedation. Further, polypharmacy may result in drug interactions (Figure 2H). THC is metabolized by the CYP family of enzymes, therefore, CYP inducers or inhibitors may alter serum levels of THC, influencing risk of impairment (58, 72). It will therefore be important for future studies to report any relevant patient medications as potential confounding factors.

### Tolerance

One of the important differences between the medical cannabis patient and those who use recreational cannabis is the pattern of THC use (e.g., intermittent vs. daily consumption). Medical cannabis patients typically manage symptoms using THC on a daily basis, which can lead to pharmacological tolerance, including tolerance to possible side effects (Figure 2J) (73–77). For example, a study of patients with multiple sclerosis did not demonstrate impairment in driving-related tasks after 4–6 weeks of daily medical cannabis treatment (when compared to their baseline without medical cannabis) (78). Notably, the one study where all participants used their daily medical cannabis up until testing day found improved performance compared to normative data (45). This suggests that patients who take medical cannabis every day may not develop the same amount of neurocognitive impairment as those who previously abstained or use infrequently.

Some of the studies evaluated in this review enrolled participants with a previous history of cannabis use (44, 45), while others enrolled cannabis naïve participants (41, 43), which may contribute to the significant heterogeneity between study results. Even within medical cannabis patients, those who use medical cannabis for persistent, chronic daily symptoms vary significantly in their use patterns from those who use to control acute and intermittent symptoms. Future clinical studies should consider THC tolerance and ensure that the duration and amount of previous THC use is specified in the eligibility criteria and evaluated when interpreting results. A standardized definition for chronic, daily medical cannabis use should be implemented in future studies. For most patients, titration and monitoring of cannabis intake typically takes 4–12 weeks to achieve an optimal therapeutic effect. The titration period depends on a number of factors (Figures 2A–C,I) including comorbidities, polypharmacy, genetics, and age (30). A research definition should account for this titration period and consider stabilization to have occurred when no further dose adjustments are required over a 2 week period. This will ultimately increase the validity and applicability to research findings. Further reviews and commentary on factors that influence impairment (Figure 2) are greatly needed.

### Limitations

Findings from this review were constrained by the limitations of the current literature. Due to the heterogeneity of the study populations, study designs and protocols, and variability in the objective testing measures between studies, we were unable to complete a meta-analysis. The lack of cognitive and motor test standardization and the inconsistent methods between studies, including the type and time of testing post-THC ingestion, precluded statistical pooling of the data. There were no standardized medical cannabis products used across studies, with each study exploring varying concentrations of THC and CBD in either smoked, vaporized, or sublingual formulations, including cannabis-based medicines such as THC:CBD oromucosal spray (Figures 2F,G). Combining findings between the included studies and coming to definitive conclusions would be premature.

An additional limitation in the literature was lack of research assessing oral THC products, including cannabis oils. Due to the known pharmacokinetic differences between ingested and inhaled THC and given that many medical cannabis patients use oral formulations, it will be important for future studies to incorporate these products in their trials. An important confounder in studies on impairment are the participants underlying medical conditions (which in these studies often included illnesses that are detrimental to neurocognitive performance). Patients baseline cognitive functioning was only described and controlled for in three of the six studies (39, 40, 43), and is important to document for future studies. Blood levels of THC and its metabolites were also not assessed in any of these studies. This was a missed opportunity to obtain a better understanding of how drug levels relate to cognitive impairment in medical cannabis users with medical doses. It would also have better enabled comparison of effects between medical and recreational cannabis users.

Finally, the literature on this topic is limited by the relatively small sample sizes of included studies. Small sample sizes may overestimate treatment effects or be insufficiently powered to detect a true difference, although some studies stated they were sufficiently powered to detect differences. Future trials would provide more robust information if they had larger sample sizes and captured data on a wider range of medical cannabis patients. Nevertheless, the trends that emerged among these medical cannabis impairment studies compared to the recreational data supports that medical cannabis patients do not have the same duration or degree of neurocognitive impairment as recreational users.

## Conclusions

This review suggests that the duration of neurocognitive impairment following inhalation or sublingual absorption of THC containing products is 4 h or less in medical cannabis patients. The results of this review are consistent with the College of Family Physicians of Canada's 2014 statement that medical cannabis patients should err on the side of caution, and delay safety sensitive activities for 3–4 h if cannabis (THC) is inhaled, 6–8 h if ingested orally, and 8 h if any euphoria is experienced (79). There are important differences between medical and recreational cannabis users that may not allow for the same conclusions to be drawn about the duration or degree of impairment within the recreational cannabis population. These differences pertain to factors including the dose of THC, method of intake, patient tolerance and intent, additional chemovars added (such as CBD) and concurrent sedative or hypnotic medication intake (Figure 2). This review suggests that neurocognitive impairment in medical cannabis patients can involve multiple neurocognitive and psychomotor domains. A summary of the main conclusions and recommendations from this review can be found in Table 5.

**Table 5:** Summary of findings.

**Summary of findings**
Neurocognitive impairment following cannabis inhalation is less than or equal to 4 h in medical cannabis patients, independent of their dosing regimen (e.g., daily, intermittent, or infrequent)
Impairment is THC dose-dependent
Acute impairment was found to be statistically significant in the following neurocognitive and psychomotor domains: • Immediate and delayed verbal recall • Processing speed • Task switching • Visual attention • Fine motor coordination • Working memory
There are several non-modifiable factors that influence duration and degree of impairment: • Comorbidities • Personal/ Family Mental Health History • Genetics and metabolism
Medical cannabis patients consume cannabis to manage symptoms and improve quality of life by optimizing the following modifiable domains: • Intent of use • Route of administration • Chemovar selection • CBD content • Dose • Tolerance • Alcohol & other sedating substances • Drug interactions
We cannot extrapolate the conclusions found in this review to recreational cannabis populations or those “medical cannabis” patients not under the guidance of a health care practitioner.

## Data Availability Statement

The original contributions presented in the study are included in the article/supplementary material, further inquiries can be directed to the corresponding author.

## Author Contributions

LE was primarily responsible for the review of published abstracts, with additional support from LL, and wrote the first draft. CM supervised the project and provided the overall intellectual leadership. All other authors contributed to revising the manuscript with additional intellectual input.

## Conflict of Interest

CM has received support for industry sponsored continuing medical education from Canopy, Medreleaf, Doja, Compass Cannabis Clinics. She has been on the scientific advisory board of Emerald Health Therapeutics, Shoppers Drug Mart, Strainprint and Resolve. She is the Medical Director of Greenleaf medical clinic, Vitality and Translational Life Sciences. She is on the Board of Directors for The Green Organic Dutchman. WP is the founder and CEO of Translational Life Sciences, an early stage science company focused on cannabinoid therapeutics. He has also been on the scientific advisory board of Medipure Pharmaceuticals and Vitality Biopharma, and in the past has been on the board of directors for Abbatis bioceuticals and on the advisory board of Vinergy Resources. AB has been a scientific advisor to Emerald Health Therapeutics, Cannevert Therapeutics, Global Cannabis Applications Corp, Medipure Pharmaceuticals, Vitality Biopharma and Hai Beverages. The remaining authors declare that the research was conducted in the absence of any commercial or financial relationships that could be construed as a potential conflict of interest.
